# Imaging Features of Hypertrophic Olivary Degeneration

**DOI:** 10.5334/jbr-btr.1065

**Published:** 2016-07-25

**Authors:** Ruth Van Eetvelde, M. Lemmerling, T. Backaert, N. Favoreel, B. Geerts, C. Sommeling, D. Hemelsoet, S. Dekeyzer

**Affiliations:** 1 University Hospital Ghent, BE; 2 AZ Sint-Lucas, BE; 3 VUB, BE; 4 Sint-Andries Hospital, Tielt, BE; 5 UZ Gent, BE; 6 UK RWTH Aachen, Duitsland

**Keywords:** Hypertrophic olivary degeneration

## Abstract

Hypertrophic olivary degeneration (HOD) is a unique form of transneuronal degeneration caused by a disruption of the dentato-rubro-olivary pathway, also known as the triangle of Guillain-Mollaret. The triangle of Guillain-Mollaret is involved in fine voluntary motor control and consists of both the inferior olivary nucleus and the red nucleus on one side and the contralateral dentate nucleus. Clinically, patients classically present with symptomatic palatal myoclonus. Typical magnetic resonance imaging findings include T2-hyperintensity and enlargement of the inferior olivary nucleus evolving over time to atrophy with residual T2-hyperintensity. In this article, we provide a case-based illustration of the anatomy of the Guillain-Mollaret-triangle and the typical imaging findings of hypertrophic olivary degeneration.

## Introduction

Hypertrophic olivary degeneration (HOD) is usually caused by a lesion along the dentate-rubro-olivary pathway, also known as the anatomic triangle of Guillain-Mollaret. The classical clinical presentation consists of palatal myoclonus. HOD can also be an incidental imaging finding in asymptomatic patients. Furthermore, in slightly less than half of patients, no causative lesion along the denta-rubro-olivary pathway can be detected. Hence, awareness of the imaging characteristics of the entity is warranted to accurately differentiate it from primary lesions in the medulla oblongata. The aim of this article is to illustrate the anatomy of the dentate-rubro-olivary pathway on MRI, illustrate the typical imaging findings and causes of HOD, and discuss its radiological differential diagnosis.

## Case Report 1

**Figure 1:**
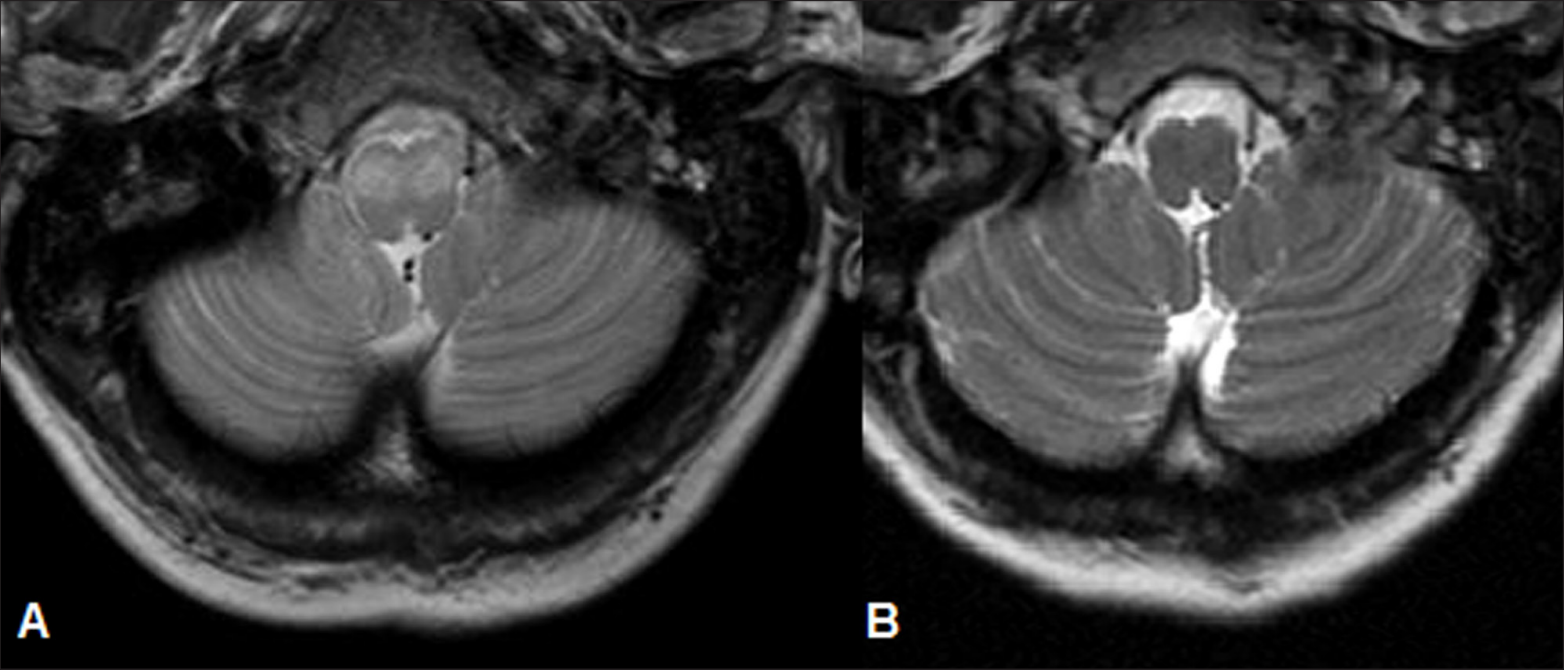
Axial T2-weighted image at the level of the medulla shows symmetric hyperintensity and expansion of the medullary olives (A). Axial T2-weighted image at the same level in the same patient but eight years earlier showed no abnormality (B).

A 55-year-old female patient consulted the neurologist because of vertigo and tinnitus. Clinical examination revealed an obvious palatal myoclonus. Magnetic resonance imaging (MRI) of the brain was performed, and T2-weighted and fluid-attenuated inversion recovery (FLAIR) images showed focal hyperintensity and expansion of both medullary olives (Figure 1). Neither enhancement was seen following contrast administration, nor restriction on diffusion-weighted imaging. Based on the clinical symptoms and radiologic findings, a diagnosis of HOD was made. The etiology for this case remains unknown since no history of infarction, hemorrhage, tumor, or trauma was present.

## Case Report 2

**Figure 2:**
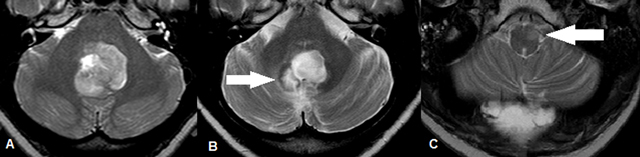
Initial MRI demonstrates a mass in the fossa posterior and fourth ventricle with heterogeneous high signal intensity on axial T2-weighted images and only moderate enhancement after injection of intravenous gadolinium (not shown) (A). MRI obtained seven months postoperatively. The axial T2-weighted image on the left shows a postoperative defect in the right dentate nucleus (white arrow) (B) and a new expansile T2- hyperintense lesion in the left inferior olivary nucleus (white arrow) (C).

A 57-year-old woman presented with vertigo. An MRI of the brain revealed a mass in the right posterior fossa (Figure 2A). The tumor was resected and an anatomopathological diagnosis of a medulloblastoma was made. A follow-up MRI obtained five months after surgery revealed a postoperative defect in the vermis immediately posterior to the fourth ventricle and extending to the right dentate nucleus, as well as a new T2-hyperintense lesion in the left inferior olivary nucleus (Figures 2B and 2C). This lesion did not enhance after contrast administration and showed no restriction on diffusion-weighted imaging.

## Case Report 3

**Figure 3:**
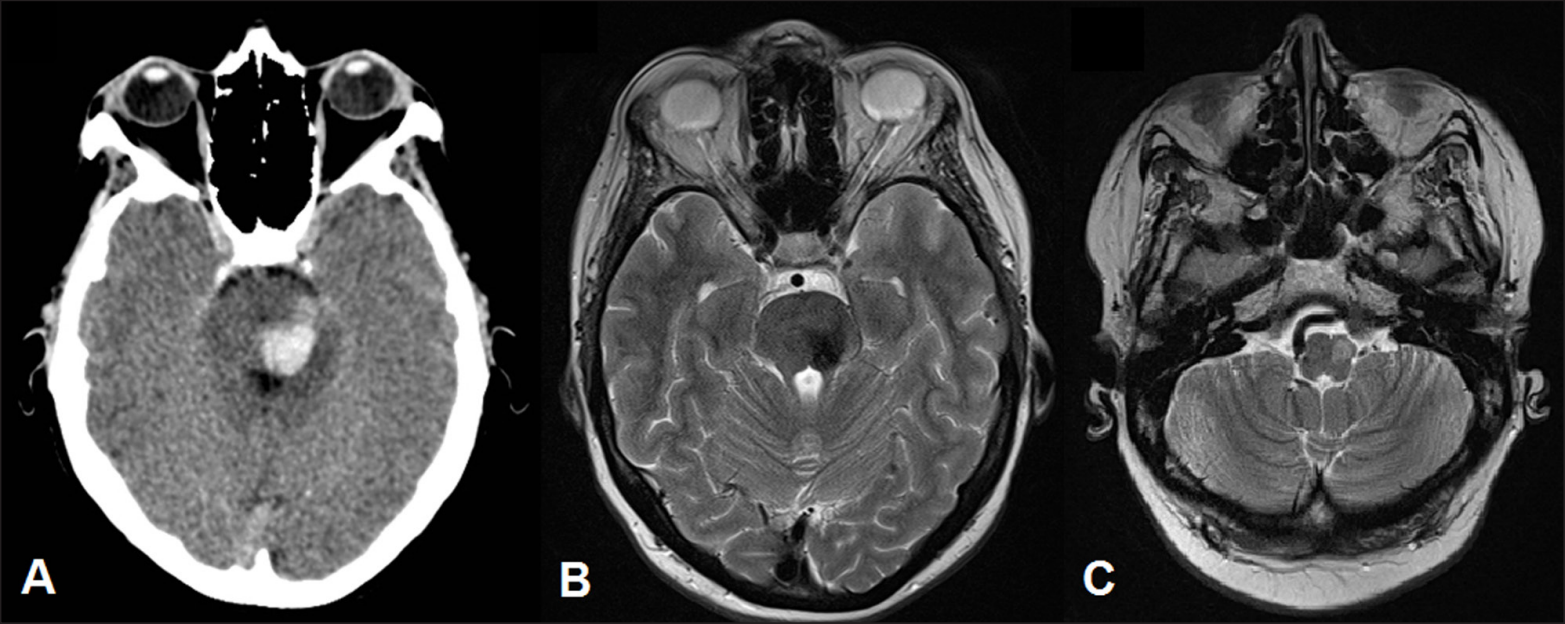
Unenhanced CT shows an acute hemorrhage in the left dorsal pontine tegmentum (A). Follow-up MRI four months later shows residual hemosiderin deposits in the area of the previous hemorrhage (B) as well as a new expansile T2-hyperintense lesion in the left inferior olivary nucleus (C).

A 43-year-old woman suffered a left pontine hypertensive hemorrhagic stroke (Figure 3A). On a follow-up MRI performed four months later, irregular T2-hypointensities could be seen in the left dorsal tegmentum of the pons, compatible with hemosiderin and ferritin deposits (Figure 3B). Furthermore, a nodular T2-hyperintense lesion could be seen in the left anterolateral part of the ventral medulla oblongata, showing no restriction and no enhancement, respectively, on diffusion and postcontrast imaging (Figure 3C). This lesion was not present on an MRI performed shortly after the hemorrhage but remained completely unchanged on a follow-up MRI performed one month later. Based on the typical location of the lesion, a diagnosis of HOD was made. At the follow-up consultation, the patient declared she intermittently experienced symptoms that could be subscribed to palatal myoclonus. However, clinical examination could not objectify these symptoms.

## Discussion

### Anatomy of the Guillain-Mollaret Triangle

**Figures 4 and 5:**
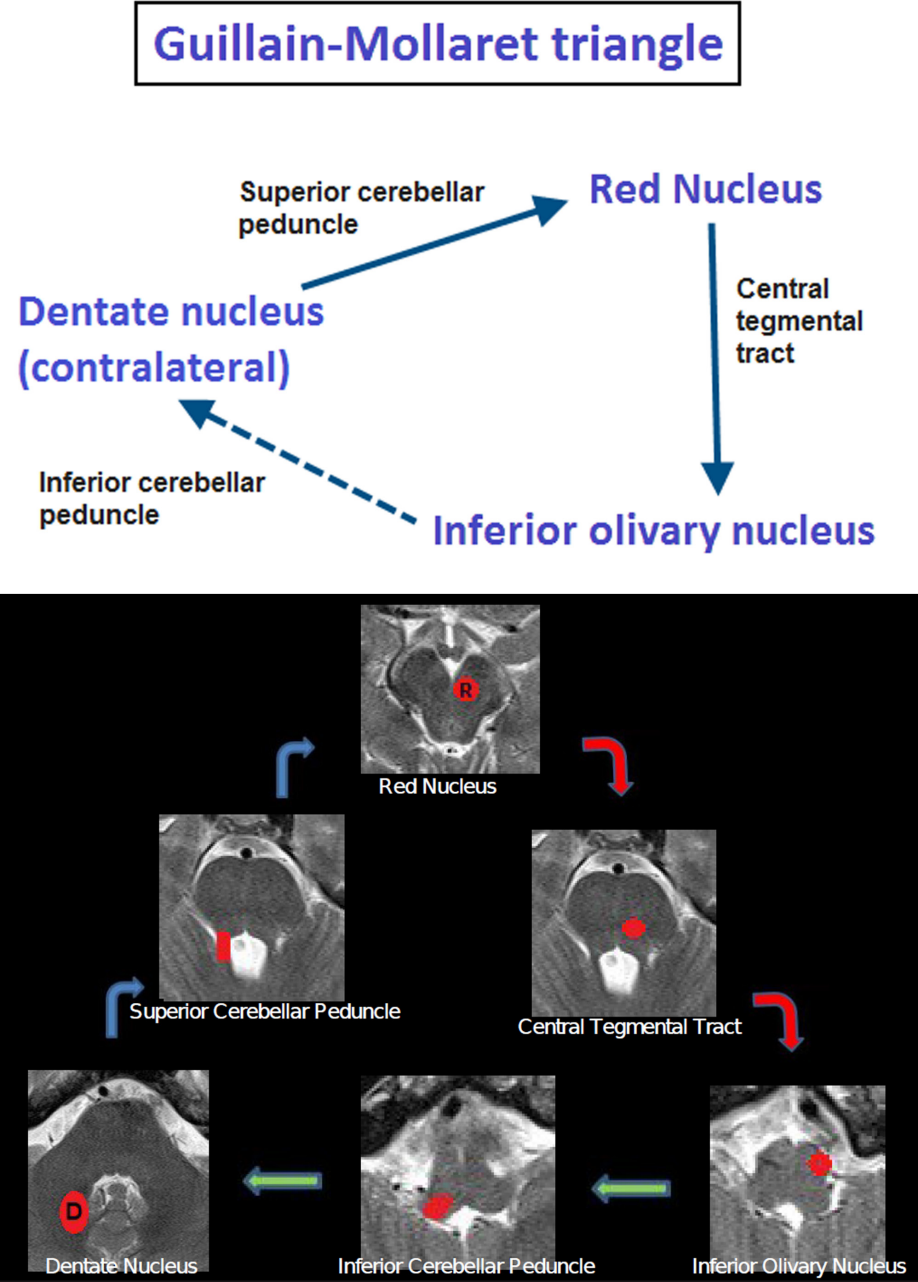
Schematic representations of the Guillain-Mollaret triangle. The contralateral dentate nucleus sends efferent fibers to the red nucleus via the superior cerebellar peduncle (*dentato-rubral pathway*). The red nucleus in turn sends efferent fibers to the inferior olivary nucleus via the central tegmental tract (*rubro-olivary pathway*). Finally, the inferior olivary nucleus sends efferent fibers the contralateral cerebellar cortex via the inferior cerebellar peduncle (*olivo-dentate pathway*), which in turn connects to the contralateral dentate nucleus, hence completing the triangle.

Hypertrophic olivary degeneration (HOD) is a rare type of neuronal degeneration caused by damage to the dentato-rubro-olivary pathway or the triangle of Guillain-Mollaret (Figures 4 and 5) [1]. The dentato-rubro-olivary pathway is a neural network involved in fine voluntary motor control and consists of the red nucleus, the ipsilateral inferior olivary nucleus, and the contralateral dentate nucleus [2]. Efferent fibers from the dentate nucleus course in the superior cerebellar peduncle and connect to the contralateral red nucleus after decussation in the brachium conjuntivum (decussation of the superior cerebellar peduncle). The red nucleus in turn sends efferent fibers through the central tegmental tract to the ipsilateral inferior olivary nucleus. The inferior olivary nucleus completes the triangle by connecting to the contralateral cerebellum via the inferior cerebellar peduncle. In this regard, it is important to notice that the fibers from the inferior olivary nucleus do not directly project to the dentate nucleus but first synapse in the cerebellar cortex via the olivocerebellar tract and then project to the dentate nucleus [3].

### Pathophysiology of Hypertrophic Olivary Degeneration

HOD only occurs with lesions involving the dentate-rubral and rubro-olivary pathways. Isolated lesions of the inferior cerebellar peduncle (olivodentate pathway) do not cause palatal myoclonus or HOD. As the olivodentate tract is an efferent tract, this is in line with the hypothesis that HOD is triggered by deafferentiation of the inferior olivary nucleus. Three patterns of HOD can be seen according to the location of the primary lesion. HOD is unilateral when the primary lesion is limited to the red nucleus or central tegmental tract, contralateral when the lesion involves the dentate nucleus or superior cerebellar peduncle, and bilateral when lesions involve the decussation of the superior cerebellar peduncle or both the superior cerebellar peduncle and the ipsilateral central tegmental tract [4,5,6].

In patient 2, a lesion in the right dentate nucleus is associated with contralateral HOD, while in patient 3, the lesion in the central tegmental tract is associated with ipsilateral HOD. In patient 1, no primary lesion could be identified. This is not unusual as in two recently published large series no lesion in the Guillain-Mollaret-triangle could be identified in up to 44 percent of patients with a radiological diagnosis of HOD [10,11]. However, a clear explanation for this finding is lacking. The authors assume that either lesions outside the Guillain-Mollaret triangle are involved or that the causative lesions in the Guillain-Mollaret-triangle are too small to be detected with MRI [10,11].

### Etiology, Symptoms, and Treatment

The etiology of HOD is broad and encompasses any lesion that interrupts the Guillain-Mollaret triangle. Focal lesions that lead to pathway interruption include, among others, ischemic infarction, tumor, demyelination, hemorrhage, trauma, and surgery [4,5,6,7,8,9]. The classical clinical presentation of HOD is palatal myoclonus, involuntary cyclical movements of the soft palate, which can present within 2 to 40 months after the initial insult [12]. Other clinical findings include dentato-rubral or Holmes’ tremor and ocular myoclonus [13,14]. The association of hypertrophic olivary degeneration with palatal myoclonus is presumed to be the result of connections between the central tegmental tract and the nucleus ambiguus [3]. The vagus nerve is involved in the control of palatal movement, and a lesion in the dentato-rubral pathway may cause loss of inhibitory control transmitted through these pathways, with palatal myoclonus and other sorts of movement disorders as a result. Not all patients with clear HOD on imaging are symptomatic however [11,15]. The symptoms are difficult to treat and rarely resolve, but successful management with benzodiazepines and carbamazepine has been reported [11,15].

### Pathology and Radiology

HOD is a unique type of transsynaptic degeneration because it leads to hypertrophy rather than atrophy. Pathologically, olivary enlargement corresponds to an unusual vacuolar degeneration of cytoplasm and an increased number of astrocytes. Goto et al. documented the sequential pathologic changes in HOD and described six stages ranging from immediate onset to several years from the primary lesion: (1) in the first 24 hours, there is no olivary change; (2) in two to seven days or more, there is degeneration of the white matter olivary capsule; (3) at approximately three weeks, there is mild olivary hypertrophy without glial reaction; (4) at approximately eight and a half months, olivary enlargement occurs with hypertrophy of both neurons and astrocytes; (5) at nine and a half months, and later, there is olivary pseudohypertrophy, in which there is neuronal dissolution with gemistocytic astrocytes; and (6) after three to four years, olivary atrophy is evident [16].

The MRI characteristics that suggest the diagnosis of HOD are high T2-signal intensity confined to the olivary nucleus or nuclei (with or without enlargement of the inferior olivary nucleus), lack of contrast enhancement or diffusion restriction, and the presence of an inciting lesion in the ipsilateral brain stem or contralateral cerebellum [17].

On MRI, three distinct stages in the development of HOD have been described [17]. Phase 1 is characterized by increased signal intensity on T2-weighted and FLAIR images without enlargement of the inferior olivary nucleus. Phase 2 typically occurs after four to six months and is characterized by both increased T2-signal intensity and enlargement of the inferior olivary nucleus. In phase 3, the enlargement resolves, which typically occurs after 10–16 months, and the inferior olivary nucleus returns to its normal volume or becomes atrophic but remains T2-hyperintense. This T2-hyperintensity remains indefinitely. In our cases, all patients presented with enlargement and increased T2-signal intensity of the involved inferior olivary nuclei. This is fitting in patients 2 and 3, seeing as in these patients HOD was detected on an MRI five months and four months after the inciting insult, respectively.

### Radiologic Differential Diagnosis

**Figure 6:**
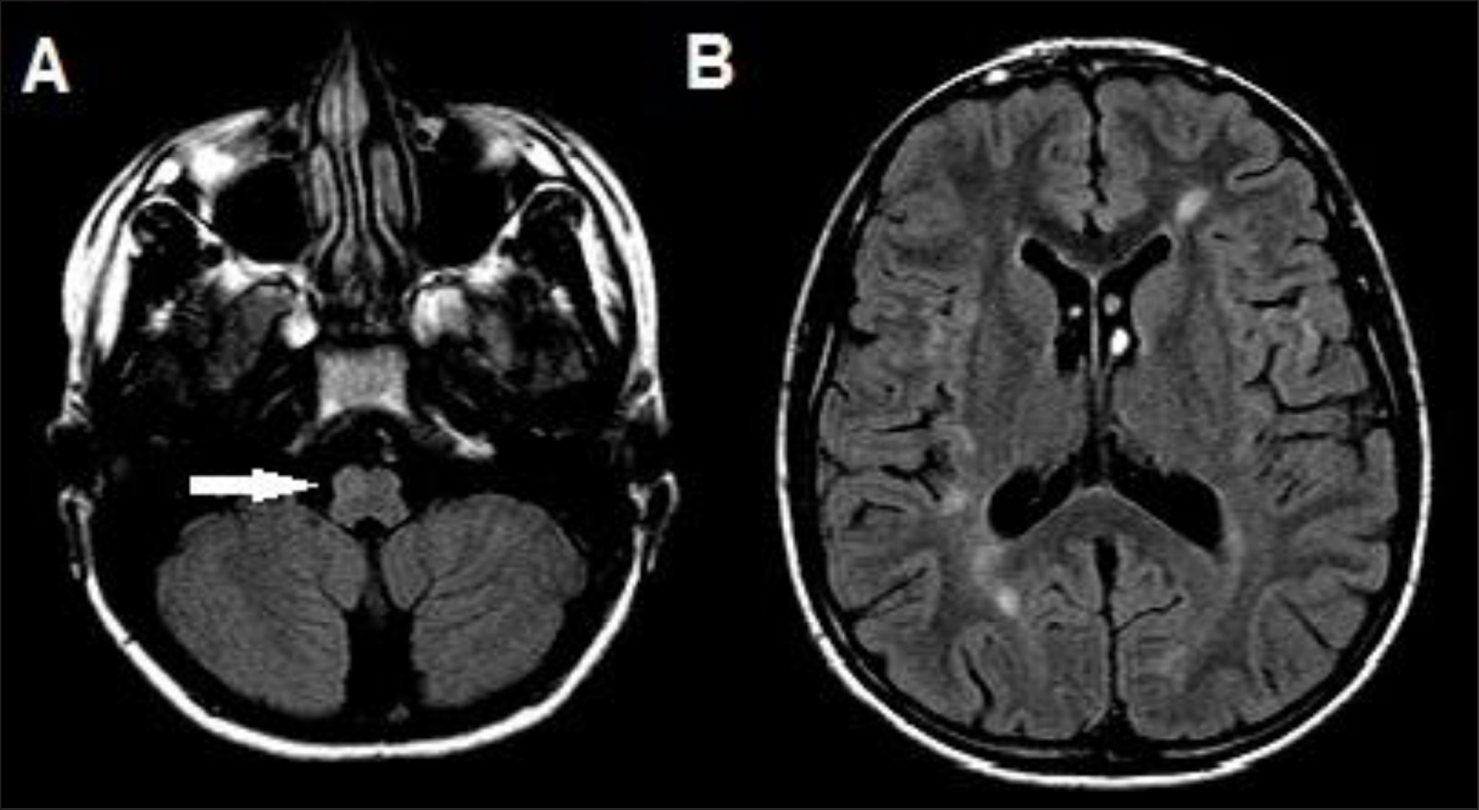
Axial FLAIR images at the level of the medulla oblongata (A) and the lateral ventricles (B) in a 36-year-old woman with established multiple sclerosis show a discrete hyperintense lesion ventrally and right-sided in the medulla oblongata (white arrow) (A). Although this lesion could reflect a hypertrophic degeneration of the right-sided inferior olivary nucleus, there were no other lesions along the Guillain-Mollaret triangle, and the patient did not exhibit symptoms typical for HOD. Due to the simultaneous presence of multiple periventricular and juxtacortical supratentoriell white matter lesions (B), a diagnosis of a demyelinating lesion was made.

The radiologic differential diagnosis includes, among others, infarction, neoplasia, demyelination, and inflammatory processes (tuberculosis, sarcoidosis, or encephalitis) (Figure 6). The absence of contrast enhancement helps in differentiating HOD from tumor, infection, and inflammation; the enlargement of the inferior olivary nucleus aids in the differential with chronic infarction or MS; and, finally, absence of diffusion restriction helps in ruling out acute infarction. Further clues to the diagnosis can be the presence of a remote lesion along the Guillain-Mollaret triangle and the fact that HOD is anatomically limited to the inferior olivary nucleus with sparing of the surrounding medullary tissue. Other radiological differential diagnoses are Wallerian degeneration, amyotrophic lateral sclerosis, and adrenoleukodystrophy in which T2-hyperintensity of/in the corticospinal tract has to be differentiated from a hyperintense inferior olivary nucleus.

## Conclusion

Hypertrophic olivary degeneration is a rare form of transsynaptic degeneration and characterized by T2-hyperintensity with or without enlargement of the inferior olivary nucleus. As patients can be asymptomatic, it can be an incidental imaging finding, and radiologists should be aware of it’s typical imaging characteristics. The absence of contrast enhancement and diffusion restriction as well as the spatial confinement of the lesion to the boundaries of the inferior olivary nucleus can help in coming to a correct radiological diagnosis. The presence of a remote lesion along the Guillain-Mollaret triangle can be a further clue to the diagnosis. The absence of lesions along the Guillain-Mollaret triangle do not rule out HOD, however, and have been described in up to 44 per cent of HOD patients.
